# *Asaia* spp. exposure for improving mosquito mass-rearing, and the effects on *Culex pipiens pipiens* vector competence for West Nile virus

**DOI:** 10.1371/journal.pone.0330703

**Published:** 2025-08-21

**Authors:** Alessandro Roman, Charlotte Linthout, Ben Raymond, Constantianus J. M. Koenraadt

**Affiliations:** 1 University of Exeter, Centre for Ecology and Conservation, Penryn, United Kingdom; 2 Wageningen University & Research, Laboratory of Entomology, Wageningen, The Netherlands; Camerino University: Universita degli Studi di Camerino, ITALY

## Abstract

Various vector control strategies are in place to reduce the spread of arthropod-borne viruses. Some of these, such as application of insecticides, are encountering operational challenges and a reduced overall effectiveness due to evolution of resistance. Alternative approaches for mosquito population control, such as the sterile insect technique, depend on efficient mass-rearing of healthy mosquitoes prior to mass-release in the field. Therefore, improving efficiency and quality of mass-rearing techniques is crucial to obtain fit mosquitoes. Previous studies have shown that Acetic Acid Bacteria of the genus *Asaia* can have a mutualistic effect on larval development in different mosquito species and can thus contribute to improved rearing output. However, whether improved performance in the larval stages may have knock-on effects in the adult stage, for example by increasing their capability to transmit arbovirus, remains unclear. Such effects may jeopardize future control efforts. We tested the effects of two *Asaia* species, *Asaia krungthepensis* and *Asaia bogorensis,* on development time and adult size under two rearing conditions: individual rearing and group rearing of *Culex pipiens* larvae. Besides investigating development and size, we also investigated whether *Asaia* spp. exposure during the larval stage can influence the vector competence of *Culex pipiens pipiens* for West Nile virus (WNV). Our work shows the potential of improving mass-rearing efficiency by employing *Asaia krungthepensis* as a mutualist for *Culex pipiens pipiens*. Importantly, this study reveals no significant increase in dissemination and transmission rate of WNV by *Culex pipiens pipiens* when inoculated with *Asaia* spp., although an increase in viral titer in the legs and the saliva was observed when the mosquitoes were inoculated with the two *Asaia* species. Interestingly, we confirmed that *Asaia* spp*.* bacteria did not establish as a permanent member of the microbiota of *Culex pipiens pipiens.* As *Asaia* spp. did not establish in adult mosquitoes, the observed change in WNV titers can be a result of indirect interactions of *Asaia* with the native *Culex pipiens pipiens* microbiome. Our results stress the importance of carefully evaluating host-symbiont interactions to avoid the potential of releasing mosquitoes with enhanced vector competence.

## Introduction

Effective mosquito control programs are needed to minimize outbreaks of mosquito-borne diseases such as malaria, chikungunya, Zika, yellow fever, dengue and Rift Valley fever [1]. The control of mosquitoes is key in interfering with the transmission cycle of these viruses, as it limits the frequency of contact between mosquitoes and humans. Various control approaches are in place such as the introduction of natural enemies (e.g., copepods) in water bodies [2,3], habitat elimination, and the application of chemical insecticides [4]. However, frequent and inappropriate use of chemical insecticides has adverse effects on populations of non-target organisms and may result in insecticide resistance in mosquito populations [5].

Novel approaches to mosquito control have been studied intensively over the past years, including the Sterile Insect Technique (SIT) and genetic modification of mosquitoes [6–8]. Both techniques are based on the mass-rearing and release of sterile males or of genetically modified males that must compete with wild-type males once they are released. However, irradiation needed for SIT increases larval and pupal mortalities and decreases adult emergence from pupae [9]. Furthermore, genetic modification of mass-produced mosquitoes may reduce mosquito fitness and sexual competitiveness in different mosquito species [10–12]. There is, therefore, a need for healthier and fitter mass-reared males. Recent studies have shown that the use of symbionts as probiotics can help to improve the fitness of mass-reared mosquitoes [13–15]. In addition, bacterial symbionts such as the obligate intracellular bacterium *Wolbachia*, can protect mosquitoes from harmful pathogens in mosquitoes and in other insects [16,17].

Possible candidates as beneficial symbionts in mosquito mass-rearing systems are bacteria of the genus *Asaia* [18–20]*. Asaia* bacteria have been identified in plants originating from tropical regions [21–23] and reported as spoilage microorganisms of fruit-flavoured bottled water [24–26]. These bacteria are of increasing interest to the scientific community because of their complex interactions with mosquito hosts. For example, Mitraka and collaborators [19] have shown an increase in the development rate of *Anopheles gambiae* mosquitoes when inoculated with *Asaia* bacteria. *Asaia* bacteria are known to inhabit various tissues within mosquito bodies, including the midgut and salivary glands [20], suggesting their potential involvement in vector competence. Therefore, Acetic Acid Bacteria of the genus *Asaia* have also been studied as potential vehicles for the expression of heterologous effector proteins that, for example, inhibit oocyst formation of *Plasmodium berghei* or activate immune responses in *Aedes aegypti* against *Dirofilaria immitis* [27,28]. In *Culicoides nubeculosus* a reduction in the abundance of *Asaia* bacteria in gut bacterial communities due to antibiotic treatment, was associated with an increased infection with Schmallenberg virus [16]. Similar changes in *Asaia* abundance in *Ae. aegypti* however, did not lead to any changes in susceptibility to Zika or chikungunya virus [16]. *Asaia* can thus have a mutualistic effect on larval development in different mosquito species and contribute to improved rearing output. However, whether addition of *Asaia* as probiotic may have knock-on effects in the adult stage, for example by increasing their capability to transmit arbovirus, remains unclear. As such effects may jeopardize mosquito control efforts, we therefore investigated the effects of *Asaia* on development of *Culex pipiens* and their capacity to transmit West Nile virus (WNV).

The first part of our work investigated the hypothesis that various bacterial species within the genus *Asaia* act as mutualists for *Culex pipiens pipiens* development when exposed to these bacteria in the rearing water, similar to our earlier observations with *Ae. aegypti* [29]. We investigated two scenarios: an individual rearing scenario, for which we studied the effects of *Asaia* spp. treatment on individually reared mosquitoes, avoiding additional effects of competition amongst larvae, and a group rearing scenario to simulate mass-rearing conditions. It is particularly important to demonstrate the potential beneficial effects of *Asaia* bacteria when provided to larvae with a complex microbiome, since mass-rearing facilities do not employ axenic production techniques. Furthermore, we investigated the effect of the *Asaia* treatments in the larval stage on the vector competence of adult *Cx. p. pipiens* females for WNV, as it is inevitable that also a small number of females would be mechanically selected for mass-release, along with the sterile males [30,31].

## Results

### Development rate of individually reared larvae

**Fig 1:**
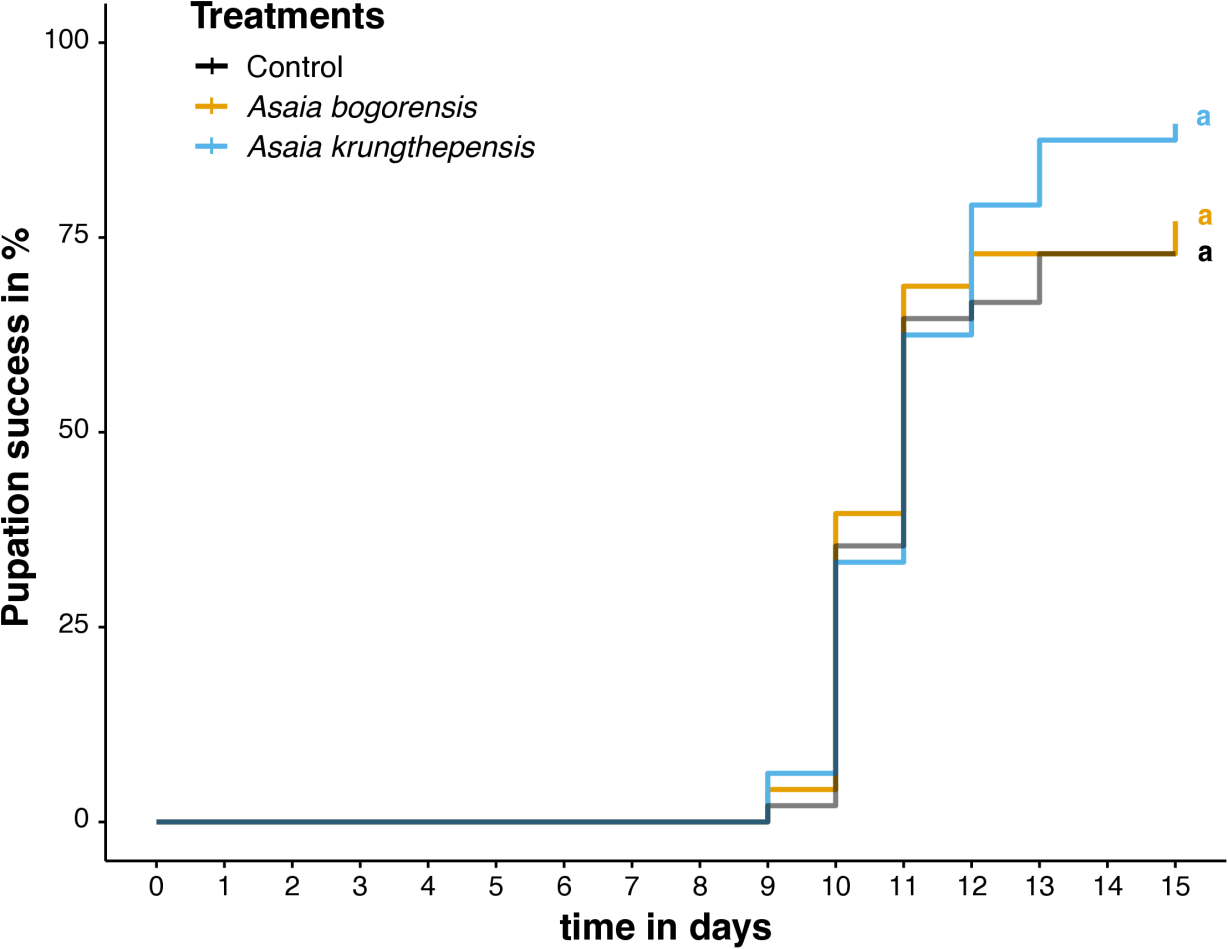
Pupation success over time for *Culex pipiens pipiens* in the individual rearing after inoculation with two different *Asaia* species. Treatments consisted of 48 biological replicates each. Differences in letters indicate significant differences between the treatments.

We hypothesized that the two species of *Asaia* act as mutualists for *Cx. p. pipiens* and reduce the time needed to reach the pupal stage. However, contrary to our hypothesis, exposure to *Asaia* spp. did not show significant effects on the development rate (Parametric model with Log-logistic error: χ^2^ = 0.62, d.f. = 2, *p* = 0.734). At day 15 of the experiment, *A. krungthepensis* inoculated larvae reached a pupation rate of 89.5%, while *A. bogorensis* inoculated larvae and the control reached rates of 77.1% and 72.9%, respectively, but these did not differ statistically (Fig 1).

### Wing length of individually reared larvae

**Fig 2:**
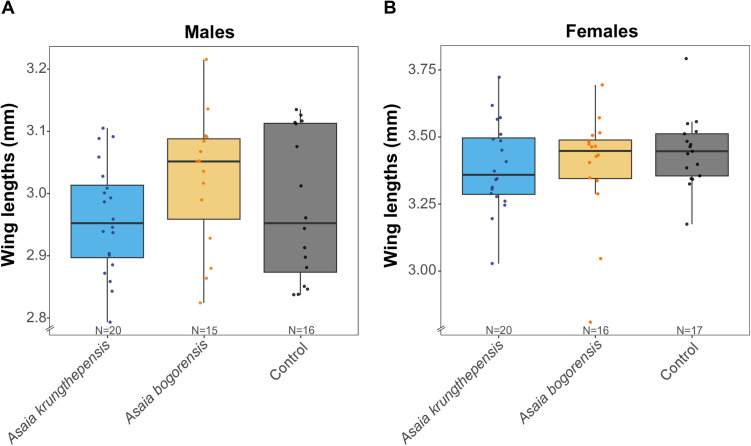
Wing lengths among *Asaia* spp. treatments and control in *Culex pipiens pipiens* in an individual rearing experiment. **A**) *Cx. p. pipiens* males and **B**) *Cx. p. pipiens* females wing length reported in millimeters.

No significant differences in wing length were observed among the treatments, in both males and females (Fig 2, one-way ANOVA: F_2, 48_ = 1.56, *p* *=* 0.227; F_2, 50_ = 0.58, *p* *=* 0.563; respectively).

### Development rate in group-reared larvae

**Fig 3:**
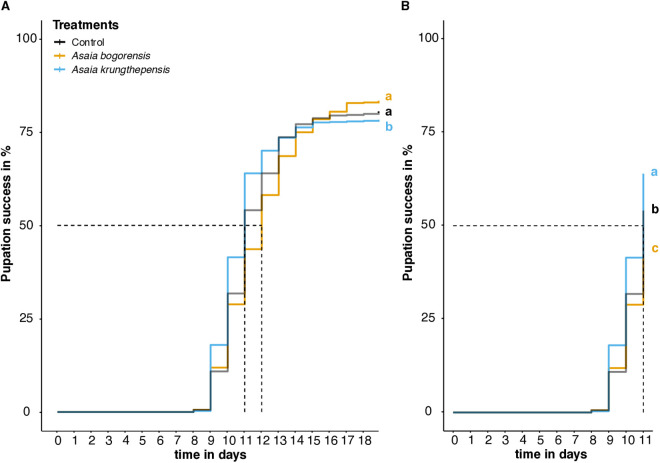
Pupation success over time for group-reared *Culex pipiens pipiens* larvae inoculated with *Asaia bogorensis*, *Asaia krungthepensis* or a control group. **A)** Full time period experiment. **B)** Graph until 50% pupation was reached by the control group. Different letters (a, b, c) indicate significant differences between the treatments. Each treatment consists of 690 larvae divided into three experimental replicates: 190 larvae in the first experimental replicate and 250 larvae in both the second and third experimental replicate. The horizontal dotted line corresponds to 50% pupation success. Vertical dotted lines correspond to the day in which the treatments reached or overcame 50% pupation.

When the entire experimental period is considered, a significant effect of the *Asaia* treatment in development rate was observed (Parametric model with Log-logistic error: χ^2^ = 13.237, d.f. = 2, *p* = 0.001). It was predominantly *A. krungthepensis* that accelerated development relative to the control larvae (time ratio = 0.966, z = −2.11, *p* = 0.035) (Fig 3A). In a mass-rearing facility, achieving a higher daily production of pupae can have significant economic value. In our experiment, the control treatment in the group-reared larvae exhibited similar results as the counterpart in the individually reared larvae, with 50% pupation (P_50_) reached around day 11 and 75% pupation by day 15 (Fig 1 vs. Fig 3A). Analysis of deviance indicated a significant effect of bacterial treatments at the timepoint in which the control group reached 50% of pupation (χ^2^ = 46.910, d.f. = 2, *p* < 0.0001), after controlling for batch-to-batch variability (χ^2^ = 44.231, d.f. = 1, *p* < 0.0001) (Fig 3B). More specifically, larvae inoculated with *A. krungthepensis* reached 64% pupation in comparison with the uninoculated larvae that reached 54% pupation, decreasing the pupation time (mean time changes = −0.291, z = −4.39, *p* < 0.0001) at day 11. Larvae exposed to *A. bogorensis* reached 44% pupation at day 11, increasing the pupation time compared to the uninoculated larvae (mean time changes = 0.164, z = 2.38, *p* = 0.018).

### Wing length in group-reared larvae

**Fig 4:**
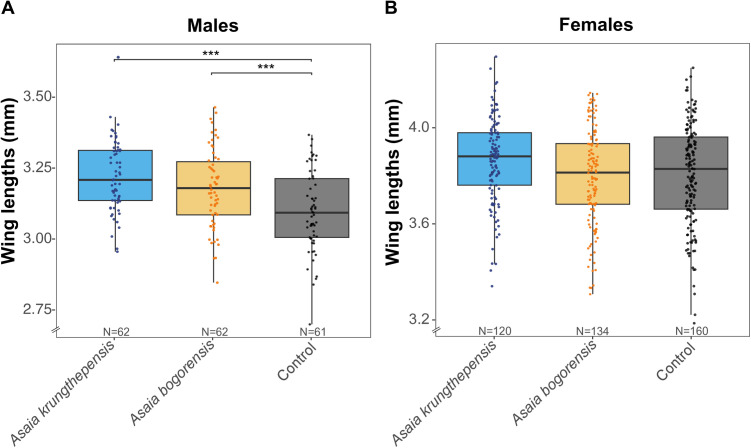
Wing lengths among *Asaia* spp. treatments and control in *Culex pipiens pipiens* males (A) and females (B) reared in a group-rearing setting. Wing length reported in millimeters. Asterisks (*) indicate significant differences (*** = p < 0.001).

The effect of *Asaia* exposure on female wing length was marginally non-significant (F_2, 409_ = 2.64, *p* = 0.073) (Fig 4). However, in males, *Asaia* exposure significantly affected wing length of the adults (F_2, 180_ = 18.39, *p* < 0.001). Specifically, both exposure to *A. krungthepensis* and to *A. bogorensis* increased male wing length compared to the control (t_180_ = 5.95, *p* < 0.0001; t_180_ = 4.01, *p* < 0.001). No differences were observed between *A. krungthepensis* and *A. bogorensis*. (t_180_ = 1.95, *p* = 0.127).

### WNV vector competence

We infected eight to twelve days old *Cx. p. pipiens* mosquitoes with WNV via an infectious blood meal. These mosquitoes emerged from the experiment with group-reared larvae and had thus been exposed to *A. bogorensis* or *A. krungthepensis* during larval development. As a control for the vector competence studies, we used *Cx. p. pipiens* mosquitoes that had not been inoculated with *Asaia* bacteria during their larval development. An average of 74% of the *Cx. p. pipiens* adult females took up a WNV-spiked blood meal with a titer ranging between 2 x 10^6^ and 1.12 x 10^7^ TCID_50_/ml for the three replicates. Due to time restrictions, not all mosquitoes that were still alive after the incubation period of 14 days, were used in the forced salivation assay (see Table 1).

**Table 1:** Overview of the feeding rate, survival rate, number of females used, and infection, dissemination and transmission rates per treatment.

Treatment	Feeding rate (%)	Survival rate (%)	Number of females used for salivation assay/Number available	Infection rate (%)	Dissemination rate (%)	Transmission rate (%)
Control	167/214 (78%)	160/167 (96%)	132/160	97/132 (73%)	52/132 (39%)	28/132 (21%)
*Asaia krungthepensis*	131/178 (69%)	120/131 (92%)	120/120	94/120 (78%)	50/120 (42%)	30/120 (25%)
*Asaia bogorensis*	173/218 (74%)	168/173 (97%)	134/168	92/134 (69%)	50/134 (37%)	30/134 (22%)

**Fig 5:**
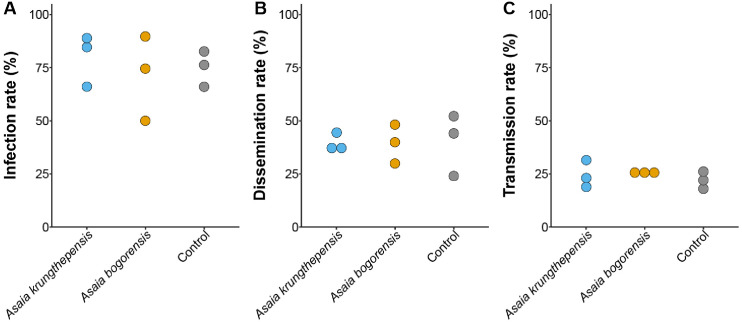
Infection (A), dissemination (B), and transmission (C) rates of WNV infected *Culex pipiens pipiens* mosquitoes. These mosquitoes were inoculated during larval development with either *Asaia bogorensis*, *Asaia krungthepensis* or neither of these *Asaia* species (control). The dots represent the rates of each individual replicate within a treatment.

Our GLMM model with wing length and titer of the blood meal as random effects included, showed that the *Asaia spp.* treatments did not have a significant effect on the infection, dissemination, and transmission rate of *Cx. p. pipiens* mosquitoes with WNV (GLMM, LTR χ^2^ = 4.414, d.f. = 2, *p* = 0.110; LRT χ^2^ = 0.271, d.f. = 2, *p* = 0.873; LRT χ^2^ = 0.955, d.f. = 2, *p* = 0.620; respectively) (Fig 5).

**Fig 6:**
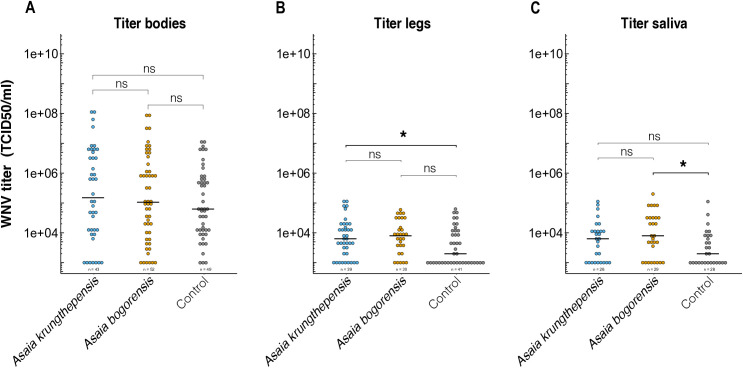
West Nile Virus (WNV) titers in body (A), legs (B) and saliva (C) samples of *Culex pipiens pipiens* adult females. These females were inoculated with *Asaia krungthepensis* or *Asaia bogorensis* bacteria during larval development. The virus titers were compared to a control treatment, which was not inoculated with *Asaia* bacteria during larval development. Asterisks (*) indicate significant differences (GLMM, * = p < 0.05), ns = not significant.

Although no effects of treatment were found concerning infection, dissemination, and transmission rates, we also investigated the effect of *Asaia* treatment on virus titer in the bodies, legs and saliva of the infected mosquitoes (Fig 6). No effects of *Asaia* spp. treatment on the viral titers in the bodies were detected (LRT: *p*= 0.751). The bacterial treatments did, however, influence the titer of WNV in mosquito legs (LRT: *p* < 0.001) after controlling for the blood meal titer effect (LRT: *p* < 0.001). Inoculation with *A. krungthepensis* significantly increased WNV titer in legs of *Cx. p. pipiens* adult females compared to the control (censReg: Estimate = 0.66, t-value = 4.07, *p* < 0.001) (Fig 6B). After controlling for experimental replicate (LRT: *p* = 0.69), bacterial treatment also significantly affected WNV titers in saliva (LRT: *p* = 0.048). Specifically, adult females derived from larvae exposed to *A. bogorensis* showed an increased titer compared to the control treatment (*censReg*: Estimate = 0.65, t-value = 2.48, *p* = 0.013) (Fig 6C).

### Detection of *Asaia* spp. in uninoculated larvae, treated and untreated female adults

**Fig 7:**
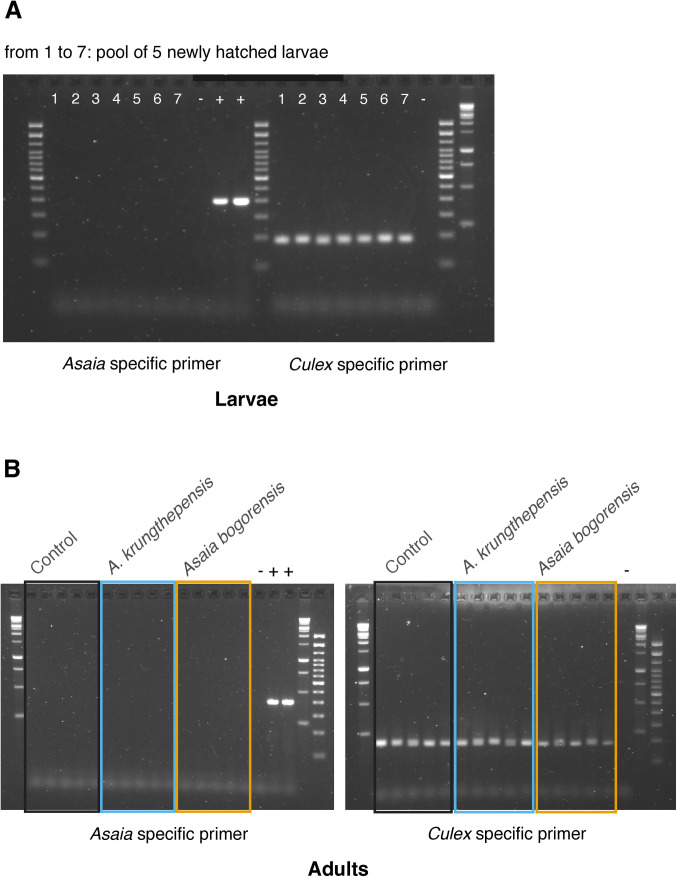
PCR detection of Genus *Asaia* in larvae and adult females. A) PCR indicates that no bacteria of the genus *Asaia* were detected in newly hatched larvae (pools of five larvae in sample 1–7). B) PCR for the detection of *Asaia*spp. in adult females. *Culex* specific primers were used as positive control of DNA extraction and PCR procedure. The symbols + and – correspond to the positive *Asaia* and the negative (water) control, respectively.

We hypothesize that an inoculum of *Asaia* spp. provided during larval development can establish as a microbiota component in mosquito larvae, and potentially be transstadially transmitted to the adult generation. *Cx. p. pipiens* larvae, hatched from mosquito egg rafts collected from the mosquito rearing showed no amplification for the genus *Asaia*. This data confirmed the absence of *Asaia* bacteria in our laboratory population of *Cx. p. pipiens* (Figure 7A). Similarly, adult females from the control did not amplify the *Asaia* DNA fragment, nor did the females that emerged from *Asaia* spp. exposed larvae (Fig 7B).

## Discussion

Fit adult mosquitoes are needed for mass-release programs to reduce mosquito populations worldwide [9,10]. Optimizing the larval diet may be one option to produce these fit mosquito populations [13–15]. However, an increase in vector competence that could be associated with more fit mosquitoes, is not favoured. In this study, we therefore investigated how inoculating *Cx. p. pipiens* first instar larvae with two different species of Acetic Acid Bacteria, belonging to the genus *Asaia*, affect fitness parameters (development rate, wing size) and vector competence for WNV.

Interestingly, the two *Asaia* spp. did not have an equal effect on development of the group-reared larvae. Larvae inoculated with *A. krungthepensis* reached 64% pupation around day 11, compared to uninoculated larvae that only reached 54% pupation and *A. bogorensis* inoculated larvae that reached only 44% pupation on the same day. Inoculation with *A. krungthepensis* therefore leads to a faster pupation. This accelerated development for *A. krungthepensis* may be advantageous for mass-rearing, as it will result in faster daily production of pupae [32,33], consequently reducing production costs [34–36]. Area-wide inundative mass-release vector control methods, such as the Sterile Insect Technique (SIT), often require releasing male mosquitoes at a ratio of 5–10 males per wild male. Recent advancements in larval rearing systems have enabled the production of approximately 100,000 male pupae per week in just 2 m² of rearing space [36]. With the increasing frequency of vector-borne disease outbreaks, the demand for sterile male mosquitoes in vector control programs is expected to rise. Therefore, reducing the development time from newly hatched larvae to pupae is critical for enhancing the weekly production capacity of mass-rearing facilities. Our findings, which show differences in pupation speed, suggest that optimizing these traits could improve production efficiency and make mass-rearing systems more cost-effective, helping meet the growing demand for sterile mosquitoes in vector control. However, its scalability and consistency under operational conditions require further consideration. While our laboratory experiments demonstrate that one *Asaia* spp. accelerates larval development, factors such as environment, rearing conditions, mosquito species, microbial competition in rearing water, and the stability of *Asaia* spp. cultures over time may influence its performance at scale. Implementing the usage of *Asaia* spp. in mass rearing facilities would necessitate pilot-scale trials and long-term assessments to validate its effectiveness under real-world conditions and to optimize protocols for routine application in mass-rearing programs.

Similar to these results, *Asaia* increases the development rate of *An. gambiae*, possibly by modulating the transcription of genes involved in the structural components of the cuticle [19]. However, in contrast with our results for *Cx. pipiens*, bacteria of the genus *Asaia* were stably associated in *An. gambiae* larvae, as they became part of the midgut microflora and were able to be transmitted transstadially [19]. Furthermore, another study demonstrated that *Anopheles stephensi* larvae deprived of *Asaia* bacteria, showed asynchrony in moulting and therefore delayed development [18]. A notable difference from our study lies in the characterization of *Asaia* within *An. stephensi*’s conventional microbiota component. Our results suggest that in *Cx. p. pipiens*, the successful colonization and transmission of *A. krungthepensis* or *A. bogorensis* may be compromised by microbial competitors present within the native microbiota, as previously shown for *Ae. aegypti* mosquitoes [29]. Although *Asaia* spp. was not detected in adult females, we acknowledge that the limited sample size precludes a definitive conclusion about persistence. In addition to microbial competition, the absence of detectable *Asaia* spp. may also reflect microbial turnover associated with metamorphosis, during which larval microbiota are frequently lost or reshaped. Additional replicates and alternative experimental approaches may help clarify the conditions required for persistence. Concerning the adult size (wing length), no differences were found neither in males nor in females within the two *Asaia* spp. treatment groups and the control when the larvae were reared individually. Similar results were obtained in *Ae. aegypti* when inoculated with *Escherichia coli* or *Asaia* sp. [14]. In the group-rearing condition, no differences were observed regarding *Cx. p. pipiens* female size, but inoculation of both *Asaia* spp. during larval development resulted in larger males compared to the uninoculated control larvae. In summary, our study suggests that particularly *A. krungthepensis* exhibits potential as larval mutualist within *Cx. p. pipiens* mosquitoes, based on both accelerated development time and larger adult males derived from both the *Asaia* spp. inoculated treatments. This species can thus serve as a candidate for vector control techniques based on mass-release of males, such as SIT. Several studies have shown that larger male body size is advantageous for survival and sperm capacity [37,38]. Male dispersal in the wild and mating success are determined by their fitness, with body size serving as a quality indicator. These aspects can directly affect longevity, the number of times a male can mate, and sperm capacity, leading to an increase or decrease of the overall efficacy of the vector control programs [38]. Our vector competence study demonstrates that, although there was no effect of treatment on the dissemination and transmission rate, we did observe increased WNV dissemination and transmission titers in *Cx. p. pipiens* adults derived from *Asaia*-exposed larvae. Interestingly, the two *Asaia* spp. influenced dissemination and transmission titers differently. Specifically, the exposure to *A. krungthepensis* significantly enhanced disseminated viral titers of mosquitoes compared to the control group, whereas *A. bogorensis* enhanced the WNV transmission titers in saliva. It is known that midgut and salivary gland barriers can be divided into the infection and escape barrier [39], and that viral infections can be limited by these barriers, mechanically as well as through antiviral immune responses. In addition, mosquito symbiotic bacteria in the gut can also have an important immunological role [40–42]. In our experiment, the transient infection with *A. krungthepensis* bacteria during larval development may alter the efficacy of the midgut barriers, but not the protective nature of the salivary gland barriers of *Cx. p. pipiens* adult females. Conversely, exposure to *A. bogorensis* bacteria resulted in *Cx. p. pipiens* females exhibiting greater WNV transmission potential compared to the non-exposed control group. In support of our findings, several studies have shown the role of mosquito gut microbiota in pathogen development and competence [43–45]. Similar to our results, the effect of *A. bogorensis* can be seen in *Anopheles stephensi* in which it can promote infection and gametogenesis of *Plasmodium berghei* [46]. Promotion of infection has been attributed to alkalization of the midgut due to proliferation of *A. bogorensis* in glucose-feeding adults [46]. Conversely, *Asaia* can stimulate the basal immunity and therefore reduce the development of malaria parasite oocysts in *An. stephensi* [47]. On the other hand, alterations in the relative abundance of *Asaia* induced by antibiotic treatment in *Aedes aegypti* did not lead to changes in susceptibility to Zika nor chikungunya virus [16]. In the *Culex pipiens* complex, the microbiota could be an important and explanatory factor for the variation in vector competence for WNV [48]. Furthermore, the reduction in abundance of the protective microbiota (*Wolbachia*) mediated by climate warming [49] can lead to increased WNV viral replication in the mosquito host [48]. Similarly, recent work provided evidence that the exposure to different bacteria during larval development can influence adult traits in *Aedes aegypti* as well as its susceptibility to systemic dissemination of dengue virus and diversity in the innate immune response [50]. In short, these studies show that it is likely that presence of or exposure to *Asaia* has effects on arbovirus replication, although reports have been conflicting regarding the directionality of this effect.

## Conclusion

Inoculation of *Cx. p. pipiens* first instar larvae with *A. krungthepensis* led to an initial acceleration of development time from newly hatched larvae to pupae. It also increased the body size of the emerging males from our mass-rearing set-up. Both the shorter development time and increased body size of male mosquitoes may improve mass-rearing conditions for *Cx. p. pipiens* mosquitoes considering mass-release based vector control applications. Vector competence studies with female *Cx. p. pipiens* mosquitoes showed that *Asaia* spp. inoculation of *Cx. p. pipiens* mosquitoes had no significant effect on infection, dissemination, and transmission rates of WNV. However, it did influence the WNV titer in the legs and the saliva of these mosquitoes, even though *Asaia* spp. bacteria were not detected in adults following larval exposure, suggesting that these bacteria did not persist or stably establish in the mosquitoes. Indirect effects of *A. bogorensis* and *A. krungthepensis* on the microbiota of the mosquitoes could be the underlying cause of these altered virus titers.

## Materials and methods

### *Culex pipiens pipiens* mosquitoes

The *Cx. p. pipiens* rearing was established in the summer of 2020 by collecting egg rafts in oviposition traps in the proximity of chicken coops across Wageningen, the Netherlands. After identifying a pool of 10 larvae per egg raft for their biotype by real-time PCR [51], the remaining larvae from the same egg raft and with biotype *pipiens* were grouped in trays to start a new rearing colony. The mosquitoes were maintained at 23°C, 60% relative humidity and a photocycle of 16:8 light:dark [52]. F19 offspring of this population was used in the experiments.

### *Asaia* bacteria cultures

Two *Asaia* isolates were used for all the experiments, *Asaia krungthepensis* isolate 1A and *Asaia bogorensis* isolate 3.10. *A. krungthepensis* isolate 1A was kindly provided by Dr. Cynara Rodovalho (FIOCRUZ, Rio de Janeiro, Brazil) and *A. bogorensis* isolate 3.10 was provided by Marc Hendriks from Wageningen University & Research. *Asaia* spp. were kept as glycerol stocks at −80°C for long-term storage. Cultures of *Asaia* spp. were grown in GLY liquid media (Glycerol 25 g/l, Yeast Extract 10 g/l, pH 5) at 30°C and 200 rpm shaking. Prior to the experiments, a single colony from each *Asaia* spp. was pre-inoculated in 3 ml GLY liquid media using 15 ml conical polypropylene centrifuge tubes (Fisherbrand™) for 8 hours at 30°C and 200 rpm, reaching an OD600 of 0.1. Next, 5 ml of sterile GLY liquid media was inoculated with 50 μl of the pre-inoculation culture and incubated for 16 hours at the same incubation conditions as mentioned previously, to reach an OD600 ⋍ 1. *Asaia* spp. growth was quantified in CFU/ml by serial dilution and plating on GLY agar plates. The targeted inoculation level at time 0 of our experiments was set at approximately 2000 CFU of *Asaia* spp. per ml of rearing water*,* using a 100-fold dilution of overnight culture, washed three times in phosphate buffer saline (PBS) and resuspended in sterile tap water.

### West Nile virus

A Dutch isolate of a passage 3 WNV lineage 2 (EMC/WNV/20TV2584/NL) was used for mosquito oral inoculation. This virus was isolated during the WNV outbreak in 2020 in the Netherlands and was kindly provided by Erasmus Medical Center (Rotterdam, the Netherlands). WNV was grown at 37°C on a monolayer of Green monkey kidney Vero E6 cells, cultured with Hepes-buffered Dulbecco’s Modified Eagle Medium (DMEM; Gibco, Carlsbad, CA, USA) and supplemented with 10% fetal bovine serum (FBS; Gibco), Gentamicin (50 μg/ml, Life technologies), Fungizone (2.5 μg/ml, Life technologies) and a combination of penicillin (100 U/ml; Sigma-Aldrich, Saint Louis, MO, USA) and streptomycin (100 μg/ml; Sigma-Aldrich, Zwijndrecht, the Netherlands) (P/S). Titrations of the WNV stock, blood meals and virus-positive samples were done on the same cell line with the same supplemented medium to determine the viral loads. This medium is hereafter referred to as DMEM Hepes complete.

### Development after exposing individually reared larvae to *Asaia* spp

Climatic conditions for experiments were as described above for the *Cx. p. pipiens* rearing. An experiment consisting of three treatment levels (*A. krungthepensis*, *A. bogorensis* and Control) was performed. *Cx. p. pipiens* eggs were hatched in sterile tap water*,* after which the larvae were transferred individually to sterile 12-well plates with lids that enable gas exchange (Cellstar™). Each well was provided with 3 ml autoclaved tap water. Each treatment consisted of 48 biological replicates, with each replicate being a single larva. Single neonate larvae were inoculated depending on the designed treatment with approximately 2000 CFU/ml of either *A. krungthepensis* or *A. bogorensis*. We provided 2.85 mg of sterilized TetraMin® Baby fish food (Tetra Werke Company, Melle, Germany) per larva, over 10 days: 0.15, 0.3, 0.6, 0.6, 0.6 and 0.6 mg on days 0, 2, 4, 6, 8 and 10, respectively. The food was prepared as a stock solution of 100 µg/µl in purified water (Milli-Q® Reference A+ System, Merck Millipore) and autoclaved. Survival and pupation were recorded daily until the end of the experiment. Additionally, adult wing length measurements were taken as an estimate of body size [53,54]. For this purpose, wings were removed and mounted on sticky tape on a glass microscope slide. Wing length was measured as the distance between the alula and the wing tip, excluding the fringe scales using a Dino-Lite Edge 5 MP digital microscope and DinoCapture 2.0 software (Dino-Lite Europe, Almere, NL). The set-up was calibrated with a slide graticule of 0.01 mm.

### Development after exposing group-reared larvae to *Asaia* spp

To simulate mass-rearing conditions, whole eggs rafts of *Cx. p. pipiens* were placed on autoclaved tap water to achieve synchronous hatching. For each treatment, one plastic 195 x 195 mm tray was used. This experiment consisted of three replicates. The first replicate consisted of ~ 190 larvae/treatment tray. The tray was filled with 570 ml sterile tap water resulting in 3 ml per larva. For the second and third replicates, we aimed for ~ 250 larvae/tray filled with 750 ml sterile tap water. In the trays designed for *A. krungthepensis* and *A. bogorensis* treatments, approximately 2000 CFU/ml following the same steps as in the individual rearing study, were added at day 0 immediately before the newly hatched larvae were transferred to the trays. Sterile TetraMin® Baby fish food was provided to obtain 0.15 mg/larva on the initial experimental day. In total, approximately 2.5 to 2.8 mg food per larva was provided during development. Survival and pupation were recorded daily until the end of the experiment. Pupae were collected in 30 ml plastic cups and separated by treatment in 30 cm cubic BugDorm-1 insect rearing cages (MegaView Science Co., Ltd. Taiwan). Emerging adults were fed *ad libitum* with 6% glucose solution and were allowed to mate for 8–12 days. For all three treatments (*A. krungthepensis*, *A. bogorensis* and Control), 8–12 days old *Cx. p. pipiens* females (n = 198 + /- 20) were used in vector competence studies. Additionally, adult wing length measurements were taken as an estimate of body size [53,54].

### Vector competence of adults exposed to *Asaia* from the group-rearing experiment

In the biosafety level 3 (BSL-3) facilities of Wageningen University, the mosquitoes were allowed to feed on a WNV spiked chicken blood meal via a Hemotek PS5 feeder system. The final virus titer of the blood meals was measured and calculated to range between 2.0*10^6^ and 1.12*10^7^ TCID_50_/ml (verified by End Point Dilution Assay, EPDA). After one hour of blood feeding, mosquitoes were anesthetized with CO_2_ and the fully engorged females were selected. These mosquitoes were placed at 23°C in plastic buckets (top diameter 12.5 cm x height 11.5 cm x bottom diameter 10.5 cm) with a 16:8 light:dark cycle for a period of 14 days. As a food source, 6% glucose solution was provided *ad libitum* via cotton wool on top of the bucket for the entire incubation period.

### Forced salivation assay

After incubation for 14 days, all females were immobilized using CO_2_. The right wing of each mosquito was kept to measure wing length. The left wing and all legs of each female were removed and collected in 1.5 ml SafeSeal Eppendorf tubes, containing 5 mm zirconium oxide beads (Next Advance, AverillPark, NY, USA), without any medium. Saliva samples were collected by inserting the proboscis into a 200 μl pipet tip, containing 5 μl of a solution of 50% FBS and 25% sugar in tap water. After 45 minutes, the body was removed and stored in a 1.5 ml SafeSeal Eppendorf tube containing 5 mm zirconium oxide beads, without any medium. The individual mosquito saliva samples were then resuspended in 55 μl DMEM Hepes complete and stored in 1.5 ml Eppendorf tubes. All individual body, leg and saliva samples were stored at −80°C until further use in the infectivity assay.

### Infectivity assay and virus titration

Frozen body and leg samples were initially processed without medium using the Bullet Blender Storm (Next Advance, USA) and the Eppendorf Minispin Plus (Eppendorf, Hamburg, Germany), following the protocol of Vogels *et al.* [55]. Next, 100 μl of fresh DMEM Hepes complete medium was added to the body samples, and 60 μl to the legs and wing samples. These were then reprocessed in the Bullet blender Storm and the Eppendorf Minispin Plus, as described by Vogels *et al.* [55]. Subsequently 30 µl of either mosquito body, legs or saliva samples were added to each well of a 96 well plate with a 90% confluent monolayer of Vero E6 cells in 60 µl DMEM Hepes complete. After incubation for two hours at 37°C, 100 μl of fresh DMEM Hepes complete medium was added to the wells. The plates were then incubated at 37°C for six days. Following this incubation period, the wells were examined for the presence or absence of the virus, based on the presence or absence of cytopathic effect (CPE). The infection, dissemination, and transmission rates were determined as the percentage of WNV-positive body, leg, or saliva samples, respectively, of the total number of mosquitoes analysed. For a subset of the body, leg and saliva samples that were scored as virus-positive, the viral titers were determined via EPDA on Vero E6 cells.

### Detection of *Asaia* spp. in uninoculated larvae, treated and untreated female adults

Polymerase Chain Reaction (PCR) was employed to confirm the presence or absence of bacteria of the genus *Asaia*. Newly hatched *Cx. p. pipiens* larvae from the mosquito rearing and emerged female adults from the three individual rearing experimental treatments were collected, and stored at −20°C. Prior to DNA extraction, all samples were washed three times in sterile PBS solution and homogenized in 180 µl cold sterile PBS using a TissueLyser II instrument (Qiagen). DNA from homogenized samples was extracted with MagBind ® HDQ blood and tissue DNA 96 Kit (Omega Bio-tek, Inc., Norcross, Georgia, USA), according to the manufacturer’s instructions. Extracted DNA was tested with specific *Asaia* primers. The primer sequences used for *Asaia* were *Asaia*Primer f (5’ – GGCGCGTAGGCGGTTTACAC) and *Asaia*Primer r (5’ – TGCGCGTTGCTTCGAATTAAACCA) [56]. Amplification of *Asaia* specific DNA results in a 400 bp PCR product. PCR settings consist of an initial denaturation step of 95°C for 5 minutes, 35 cycles of 94°C for 30 sec, 55°C for 45 sec, 72°C for 30 sec and a final extension at 72°C for 7 minutes.

To validate the DNA extraction and the PCR, also the CQ11 microsatellite-specific primers for *Culex pipiens* were used: CQ11F2 (5’ – GATCCTAGCAAGCGAGAAC) and pipCQ11 r (5’ – CATGTTGAGCTTCGGTGAA) [57]. For CQ11 the PCR conditions were set as follows: 95°C for 5 min initial denaturation step, 40 cycles of 94°C for 30 sec, 54°C for 30 sec, 72°C for 40 sec, and a final extension at 72°C for 5 minutes. We tested seven pools of five newly hatched larvae per pool for presence/absence of *Asaia* at the beginning of the experiment, prior to inoculating the larvae with the bacteria. Furthermore, we evaluated the establishment of *Asaia* in adults by testing five individual females per treatment. DNA extracted from overnight cultures of *A. krungthepensis* and *A. bogorensis* was used as positive control for *Asaia*-specific primers. PCR products were run on a 2% agarose gel for 60 min at 80 V.

### Statistical analysis

All experimental data were analysed in RStudio version 2022.07.2 [58].

#### Individual rearing experiment.

Data on time to pupation were analysed using parametric models with Log-logistic errors in the *Survival* package [59] with bacterial treatment (factor with three levels, i.e., Control and the two *Asaia* spp.; *A. krungthepensis* and *A. bogorensis*) as an explanatory variable. Graphical check of the linearity of the predictors, and model fit with Cox-Snell residual analysis were performed. We also used the Akaike information criterion (AIC) to compare Log-logistic models to other error structures or analysis approaches (e.g., non-parametric models). Histograms, Shapiro-Wilk tests and Fligner tests were used to explore wing length of both male and female mosquitoes. Differences in wing lengths between the treatments for the individual rearing experiment were analysed by one-way ANOVA *aov* function [58].

#### Vector competence experiment.

Time-to-pupation was analysed using parametric models with Log-logistic errors in the Survival package [59] with bacterial treatment (factor with three levels, i.e., Control, *A. krungthepensis* and *A. bogorensis*) and experimental replicate as explanatory variable (experimental replicate was included first in the model to control for variability across batches, followed by treatment to evaluate its effect independently of batch differences). Additionally, the pupation time differences among the bacterial treatments were examined once more at the timepoint when the uninoculated control treatment had achieved 50% pupation, using parametric models with Gaussian errors and the same explanatory variables. Mosquito mass-production benefits from a faster P50 (pupation 50%) time [34]. Graphical check of the linearity of the predictors, and residual analysis were performed. The Akaike information criterion (AIC) and Bayesian information criterion (BIC) were used to compare Log-logistic models to other error structures or analysis approaches (e.g., non-parametric models). Male and female wing length measurements for the vector competence experiment were analysed with a linear mixed-effects model fitted by maximum likelihood (ML), with bacterial treatment as explanatory variable and experimental replicate number as random effect [60]. Outlier values were checked and eventually removed by using the Grubb test included in the package *outliers* [61]. Model assumptions were checked with graphical analyses of error distribution. When a significant effect of bacterial treatment was found, estimated marginal means were calculated for pairwise comparison, employing the *Emmeans* package version 1.8.8 [62].

To assess the effects of treatment (*A. krungthepensis*, *A. bogorensis* and control) during the larval stage, while controlling for the wing length of the adult females, and the titer of the infectious blood meal on WNV infection, dissemination and transmission rates, Generalized Linear Mixed-Effects Models (GLMM) with binomial distribution were performed, using the *lme4* package [60]. Infection, dissemination, and transmission rates (dependent variables) were calculated, respectively, by dividing the number of female mosquitoes with infected bodies, infected legs or infected saliva by the total number of female mosquitoes in the respective treatment.

The effect of treatment on viral titers in body, leg and saliva, were analysed with Censored Regression (Tobit) Models in RStudio using the *censReg* package version 0.5–36 [63]. By using this package, we can deal with WNV-positive samples that have titer values below the detection limit of 2000 TCID_50_/ml by setting them at 1000 in the left censored model formula. Bacterial treatment and blood-meal titer were employed as explanatory variables. All the analyses were carried out on the log10 values of the titers.

## Supporting information

S1 Data Raw image. (PDF)

## References

[1] Weaver SC, Reisen WK. Present and future arboviral threats. Antiviral Res. 2010;85(2):328–45. doi: 10.1016/j.antiviral.2009.10.008 19857523 PMC2815176

[2] Rey JR, O’Connell S, Suárez S, Menéndez Z, Lounibos LP, Byer G. Laboratory and field studies of Macrocyclops albidus (Crustacea: Copepoda) for biological control of mosquitoes in artificial containers in a subtropical environment. J Vector Ecol. 2004;29(1):124–34. 15266749

[3] Brian KH. The use of predacious copepods for controlling dengue and other vectors. WHO SEARO. 1996;20:93–8.

[4] Shaukat MA, Ali S, Saddiq B, Hassan MW, Ahmad A, Kamran M. Effective mechanisms to control mosquito borne diseases: a review. Am J Clin Neurol Neurosurg. 2019;4:21–30.

[5] Karunamoorthi K, Sabesan S. Insecticide resistance in insect vectors of disease with special reference to mosquitoes: a potential threat to global public health. Health Scope. 2013;2(1):4–18.

[6] Harris AF, McKemey AR, Nimmo D, Curtis Z, Black I, Morgan SA, et al. Successful suppression of a field mosquito population by sustained release of engineered male mosquitoes. Nat Biotechnol. 2012;30(9):828–30. doi: 10.1038/nbt.2350 22965050

[7] Tur C, Almenar D, Benlloch-Navarro S, Argilés-Herrero R, Zacarés M, Dalmau V, et al. Sterile Insect Technique in an Integrated Vector Management Program against Tiger Mosquito Aedes albopictus in the Valencia Region (Spain): Operating Procedures and Quality Control Parameters. Insects. 2021;12(3):272. doi: 10.3390/insects12030272 33807092 PMC8004901

[8] Oliva CF, Jacquet M, Gilles J, Lemperiere G, Maquart P-O, Quilici S, et al. The sterile insect technique for controlling populations of Aedes albopictus (Diptera: Culicidae) on Reunion Island: mating vigour of sterilized males. PLoS One. 2012;7(11):e49414. doi: 10.1371/journal.pone.0049414 23185329 PMC3504010

[9] Hasaballah AI. Impact of gamma irradiation on the development and reproduction of Culex pipiens (Diptera; Culicidae). Int J Radiat Biol. 2018;94(9):844–9. doi: 10.1080/09553002.2018.1490040 29913104

[10] Catteruccia F, Godfray HCJ, Crisanti A. Impact of genetic manipulation on the fitness of Anopheles stephensi mosquitoes. Science. 2003;299(5610):1225–7. doi: 10.1126/science.1081453 12595691

[11] Yao FA, Millogo A-A, Epopa PS, North A, Noulin F, Dao K, et al. Mark-release-recapture experiment in Burkina Faso demonstrates reduced fitness and dispersal of genetically-modified sterile malaria mosquitoes. Nat Commun. 2022;13(1):796. doi: 10.1038/s41467-022-28419-0 35145082 PMC8831579

[12] Bargielowski I, Nimmo D, Alphey L, Koella JC. Comparison of life history characteristics of the genetically modified OX513A line and a wild type strain of Aedes aegypti. PLoS One. 2011;6(6):e20699. doi: 10.1371/journal.pone.0020699 21698096 PMC3117796

[13] Coon KL, Vogel KJ, Brown MR, Strand MR. Mosquitoes rely on their gut microbiota for development. Mol Ecol. 2014;23(11):2727–39. doi: 10.1111/mec.12771 24766707 PMC4083365

[14] Souza RS, Virginio F, Riback TIS, Suesdek L, Barufi JB, Genta FA. Microorganism-Based Larval Diets Affect Mosquito Development, Size and Nutritional Reserves in the Yellow Fever Mosquito *Aedes aegypti* (Diptera: Culicidae). Front Physiol. 2019;10:152. doi: 10.3389/fphys.2019.00152 31024326 PMC6465640

[15] Hamden H, Guerfali MM, Fadhl S, Saidi M, Chevrier C. Fitness improvement of mass-reared sterile males of Ceratitis capitata (Vienna 8 strain) (Diptera: Tephritidae) after gut enrichment with probiotics. J Econ Entomol. 2013;106(2):641–7. doi: 10.1603/ec12362 23786049

[16] Möhlmann TWR, Vogels CBF, Goertz GP, Pijlman GP, ter Braak CJF, te Beest DE. Impact of gut bacteria on the infection and transmission of pathogenic arboviruses by biting midges and mosquitoes. Microb Ecol. 2020;80(3):703–17. 32462391 10.1007/s00248-020-01517-6 PMC7476999

[17] Bian G, Xu Y, Lu P, Xie Y, Xi Z. The endosymbiotic bacterium Wolbachia induces resistance to dengue virus in Aedes aegypti. PLoS Pathog. 2010;6(4):e1000833. doi: 10.1371/journal.ppat.1000833 20368968 PMC2848556

[18] Chouaia B, Rossi P, Epis S, Mosca M, Ricci I, Damiani C, et al. Delayed larval development in Anopheles mosquitoes deprived of Asaia bacterial symbionts. BMC Microbiol. 2012;12(Suppl 1):S2. 10.1186/1471-2180-12-S1-S2 PMC3287513 22375964

[19] Mitraka E, Stathopoulos S, Siden-Kiamos I, Christophides GK, Louis C. Asaia accelerates larval development of Anopheles gambiae. Pathog Glob Health. 2013;107(6):305–11. doi: 10.1179/2047773213Y.0000000106 24091152 PMC4001610

[20] Favia G, Ricci I, Damiani C, Raddadi N, Crotti E, Marzorati M, et al. Bacteria of the genus Asaia stably associate with Anopheles stephensi, an Asian malarial mosquito vector. Proc Natl Acad Sci U S A. 2007;104(21):9047–51. doi: 10.1073/pnas.0610451104 17502606 PMC1885625

[21] Katsura K, Kawasaki H, Potacharoen W, Saono S, Seki T, Yamada Y, et al. Asaia siamensis sp. nov., an acetic acid bacterium in the alpha-proteobacteria. Int J Syst Evol Microbiol. 2001;51(Pt 2):559–63. doi: 10.1099/00207713-51-2-559 11321102

[22] Yamada Y, Katsura K, Kawasaki H, Widyastuti Y, Saono S, Seki T. Asaia bogorensis gen. nov., sp. nov., an unusual acetic acid bacterium in the alpha-Proteobacteria. Int J Sys Evol Microbiol. 2000;50(2):823–9. 10.1099/00207713-50-2-823 10758893

[23] Yukphan P, Potacharoen W, Tanasupawat S, Tanticharoen M, Yamada Y. Asaia krungthepensis sp. nov., an acetic acid bacterium in the alpha-Proteobacteria. Int J Syst Evol Microbiol. 2004;54(Pt 2):313–6. doi: 10.1099/ijs.0.02734-0 15023938

[24] Moore JE, McCalmont M, Xu J, Millar BC, Heaney N. Asaia sp., an unusual spoilage organism of fruit-flavored bottled water. Appl Environ Microbiol. 2002;68(8):4130–1. 12147519 10.1128/AEM.68.8.4130-4131.2002 PMC123994

[25] Kregiel D, Rygala A, Libudzisz Z, Walczak P, Oltuszak-Walczak E. Asaia lannensis–the spoilage acetic acid bacteria isolated from strawberry-flavored bottled water in Poland. Food Control. 2012;26(1):147–50. doi: 10.1016/j.foodcont.2012.01.020

[26] Horsáková I, Voldřich M, Čeřovský M, Sedláčková P, Šicnerová P, Ulbrich P. Asaia sp. as a Bacterium Decaying the Packaged Still Fruit Beverages. Czech J Food Sci. 2009;27(Special Issue 1):S362–5. doi: 10.17221/970-cjfs

[27] Bongio NJ, Lampe DJ. Inhibition of Plasmodium berghei Development in Mosquitoes by Effector Proteins Secreted from Asaia sp. Bacteria Using a Novel Native Secretion Signal. PLoS One. 2015;10(12):e0143541. doi: 10.1371/journal.pone.0143541 26636338 PMC4670117

[28] Epis S, Varotto-Boccazzi I, Crotti E, Damiani C, Giovati L, Mandrioli M, et al. Chimeric symbionts expressing a Wolbachia protein stimulate mosquito immunity and inhibit filarial parasite development. Commun Biol. 2020;3(1):105. doi: 10.1038/s42003-020-0835-2 32144396 PMC7060271

[29] Roman A, Koenraadt CJM, Raymond B. Asaia spp. accelerate development of the yellow fever mosquito, Aedes aegypti, via interactions with the vertically transmitted larval microbiome. J Appl Microbiol. 2024;135(11):lxae261. 10.1093/jambio/lxae261 39419784

[30] Zheng X, Zhang D, Li Y, Yang C, Wu Y, Liang X, et al. Incompatible and sterile insect techniques combined eliminate mosquitoes. Nature. 2019;572(7767):56–61. doi: 10.1038/s41586-019-1407-9 31316207

[31] Aldridge RL, Kline J, Coburn JM, Britch SC, Boardman L, Hahn DA, et al. Gamma-Irradiation Reduces Survivorship, Feeding Behavior, and Oviposition of Female Aedes aegypti. J Am Mosq Control Assoc. 2020;36(3):152–60. doi: 10.2987/20-6957.1 33600583

[32] Robinson AS, Knols BGJ, Voigt G, Hendrichs J. Conceptual framework and rationale. Malar J. 2009;8 Suppl 2(Suppl 2):S1. doi: 10.1186/1475-2875-8-S2-S1 19917070 PMC2777322

[33] Dyck V, Reyes Flores J, Vreysen M, Regidor Fernandez E, Teruya T, Barnes B. Management of area-wide integrated pest management programmes that integrate the Sterile Insect Technique. Sterile insect technique: principles and practice in area-wide integrated pest management. Dordrecht: Springer Netherlands; 2005. p. 525–45.

[34] Mamai W, Bimbile-Somda NS, Maiga H, Juarez JG, Muosa ZAI, Ali AB, et al. Optimization of mosquito egg production under mass rearing setting: effects of cage volume, blood meal source and adult population density for the malaria vector, Anopheles arabiensis. Malar J. 2017;16(1):41. doi: 10.1186/s12936-017-1685-3 28118825 PMC5260048

[35] Balestrino F, Benedict MQ, Gilles JRL. A new larval tray and rack system for improved mosquito mass rearing. J Med Entomol. 2012;49(3):595–605. doi: 10.1603/me11188 22679867

[36] Balestrino F, Puggioli A, Gilles JRL, Bellini R. Validation of a new larval rearing unit for Aedes albopictus (Diptera: Culicidae) mass rearing. PLoS One. 2014;9(3):e91914. doi: 10.1371/journal.pone.0091914 24647347 PMC3960149

[37] Reisen W, Milby M, Bock M. The effects of immature stress on selected events in the life history of Culex tarsalis. Mosq News. 1984;44(3):385–95.

[38] Ponlawat A, Harrington LC. Age and body size influence male sperm capacity of the dengue vector Aedes aegypti (Diptera: Culicidae). J Med Entomol. 2007;44(3):422–6. doi: 10.1603/0022-2585(2007)44[422:aabsim]2.0.co;2 17547226

[39] Kenney JL, Brault AC. The role of environmental, virological and vector interactions in dictating biological transmission of arthropod-borne viruses by mosquitoes. Adv Virus Res. 2014;89:39–83. doi: 10.1016/B978-0-12-800172-1.00002-1 24751194

[40] Li M, Zhou Y, Cheng J, Wang Y, Lan C, Shen Y. Response of the mosquito immune system and symbiotic bacteria to pathogen infection. Parasit Vectors. 2024;17(1):69. doi: 10.1186/s13071-024-06161-4 38368353 PMC10874582

[41] Hegde S, Rasgon JL, Hughes GL. The microbiome modulates arbovirus transmission in mosquitoes. Curr Opin Virol. 2015;15:97–102. doi: 10.1016/j.coviro.2015.08.011 26363996 PMC5731638

[42] Jupatanakul N, Sim S, Dimopoulos G. The insect microbiome modulates vector competence for arboviruses. Viruses. 2014;6(11):4294–313. doi: 10.3390/v6114294 25393895 PMC4246223

[43] Shi H, Yu X, Cheng G. Impact of the microbiome on mosquito-borne diseases. Protein Cell. 2023;14(10):743–61. doi: 10.1093/procel/pwad021 37186167 PMC10599646

[44] Cansado-Utrilla C, Zhao SY, McCall PJ, Coon KL, Hughes GL. The microbiome and mosquito vectorial capacity: rich potential for discovery and translation. Microbiome. 2021;9(1):111. doi: 10.1186/s40168-021-01073-2 34006334 PMC8132434

[45] Ramirez JL, Souza-Neto J, Torres Cosme R, Rovira J, Ortiz A, Pascale JM, et al. Reciprocal tripartite interactions between the Aedes aegypti midgut microbiota, innate immune system and dengue virus influences vector competence. PLoS Negl Trop Dis. 2012;6(3):e1561. doi: 10.1371/journal.pntd.0001561 22413032 PMC3295821

[46] Wang M, An Y, Gao L, Dong S, Zhou X, Feng Y, et al. Glucose-mediated proliferation of a gut commensal bacterium promotes Plasmodium infection by increasing mosquito midgut pH. Cell Rep. 2021;35(3):108992. doi: 10.1016/j.celrep.2021.108992 33882310 PMC8116483

[47] Cappelli A, Damiani C, Mancini MV, Valzano M, Rossi P, Serrao A, et al. Asaia Activates Immune Genes in Mosquito Eliciting an Anti-Plasmodium Response: Implications in Malaria Control. Front Genet. 2019;10:836. doi: 10.3389/fgene.2019.00836 31608103 PMC6774264

[48] Novakova E, Woodhams DC, Rodríguez-Ruano SM, Brucker RM, Leff JW, Maharaj A, et al. Mosquito Microbiome Dynamics, a Background for Prevalence and Seasonality of West Nile Virus. Front Microbiol. 2017;8:526. doi: 10.3389/fmicb.2017.00526 28421042 PMC5378795

[49] Dohm DJ, O’Guinn ML, Turell MJ. Effect of environmental temperature on the ability of Culex pipiens (Diptera: Culicidae) to transmit West Nile virus. J Med Entomol. 2002;39(1):221–5. doi: 10.1603/0022-2585-39.1.221 11931261

[50] Dickson LB, Jiolle D, Minard G, Moltini-Conclois I, Volant S, Ghozlane A, et al. Carryover effects of larval exposure to different environmental bacteria drive adult trait variation in a mosquito vector. Sci Adv. 2017;3(8):e1700585. 10.1126/sciadv.1700585 PMC5559213 28835919

[51] Vogels CBF, van de Peppel LJJ, van Vliet AJH, Westenberg M, Ibañez-Justicia A, Stroo A, et al. Winter Activity and Aboveground Hybridization Between the Two Biotypes of the West Nile Virus Vector Culex pipiens. Vector Borne Zoonotic Dis. 2015;15(10):619–26. doi: 10.1089/vbz.2015.1820 26394124

[52] Vogels CBF, Fros JJ, Göertz GP, Pijlman GP, Koenraadt CJM. Vector competence of northern European Culex pipiens biotypes and hybrids for West Nile virus is differentially affected by temperature. Parasit Vectors. 2016;9(1):393. doi: 10.1186/s13071-016-1677-0 27388451 PMC4937539

[53] Nasci RS. Relationship of wing length to adult dry weight in several mosquito species (Diptera: Culicidae). J Med Entomol. 1990;27(4):716–9. doi: 10.1093/jmedent/27.4.716 2388250

[54] Armbruster P, Hutchinson RA. Pupal mass and wing length as indicators of fecundity in Aedes albopictus and Aedes geniculatus (Diptera: Culicidae). J Med Entomol. 2002;39(4):699–704. 12144308 10.1603/0022-2585-39.4.699

[55] Vogels CBF, Göertz GP, Pijlman GP, Koenraadt CJM. Vector competence of northern and southern European Culex pipiens pipiens mosquitoes for West Nile virus across a gradient of temperatures. Med Vet Entomol. 2017;31(4):358–64. doi: 10.1111/mve.12251 28752627

[56] Ojha A, Zhang W. A comparative study of microbial community and dynamics of Asaia in the brown planthopper from susceptible and resistant rice varieties. BMC Microbiol. 2019;19(1):139. doi: 10.1186/s12866-019-1512-9 31234788 PMC6591912

[57] Bahnck CM, Fonseca DM. Rapid assay to identify the two genetic forms of Culex (Culex) pipiens L. (Diptera: Culicidae) and hybrid populations. Am J Trop Med Hyg. 2006;75(2):251–5. 16896127

[58] R Core Team. R: A language and environment for statistical computing. Vienna, Austria: R Foundation for Statistical Computing; 2022. Available from: http://www.R-project.org/.

[59] Therneau TM. A Package for Survival Analysis in R. 2022. Available from: https://CRAN.R-project.org/package=survival.

[60] Bates D, Mächler M, Bolker B, Walker S. Fitting linear mixed-effects models using lme4. arXiv preprint arXiv:14065823. 2015.

[61] Komsta L. *outliers*: Tests for Outliers. R package version 0.15 ed. 2022. Available from: https://cran.r-project.org/web/packages/outliers/.

[62] Lenth RV. *emmeans*: estimated marginal means, aka least-squares means. R package version 1.9.0 ed. 2023. Available from: https://CRAN.R-project.org/package=emmeans.

[63] Arne Henningsen. *censReg*: Censored Regression (Tobit) Models. 2022. Available from: https://cran.r-project.org/web/packages/censReg/.

