# Supplementary material for: Asaia spp. exposure for improving mosquito mass-rearing, and the effects on Culex pipiens pipiens vector competence for West Nile virus
Source: PLoS One. 2025 Aug 21;20(8):e0330703. doi: 10.1371/journal.pone.0330703 (PMC12370026; doi:10.1371/journal.pone.0330703)

Fig 7. A) Each sample consists of a pool of 5 larvae.

From left to right:

PCR with *Asaia* specific primers (*Asaia*Primer f (5’ - GGCGCGTAGGCGGTTTACAC) and *Asaia*Primer r (5’ - TGCGCGTTGCTTCGAATTAAACCA)):

- Promega 1kb DNA ladder
- Sample1
- Sample2
- Sample3
- Sample4
- Sample5
- Sample6
- Sample7
- Water (negative control)
- *Asaia krungthepensis*
- *Asaia bogorensis*

PCR with *Cx. Pipiens* specific primers (CQ11F2 (5’ - GATCCTAGCAAGCGAGAAC) and pipCQ11 r (5’ - CATGTTGAGCTTCGGTGAA)):

- Promega 1kb DNA ladder
- Sample1
- Sample2
- Sample3
- Sample4
- Sample5
- Sample6
- Sample7
- Water (negative control)
- Promega 1kb DNA ladder
- Invitrogen 100bp DNA ladder

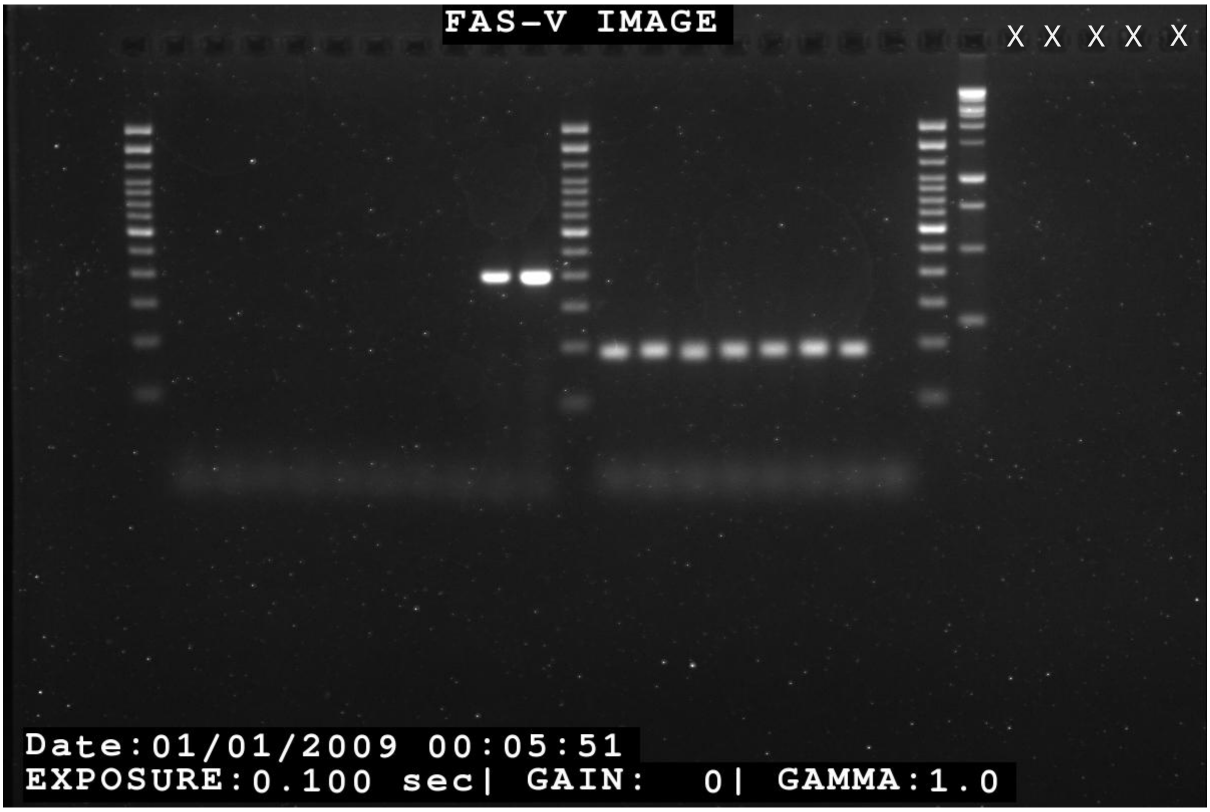

Fig 7.B) Left panel: PCR for the detection of *Asaia* spp. in adult females. *Culex* specific primers were used as positive control of DNA extraction and PCR procedure.

From left to right:

PCR with *Asaia* specific primers (*Asaia*Primer f (5' - GGCGCGTAGGCGGTTTACAC) and *Asaia*Primer r (5' - TGCGCGTTGCTTCGAATTAAACCA)):

- Promega 1kb DNA ladder
- Female Control sample1
- Female Control sample2
- Female Control sample3
- Female Control sample4
- Female Control sample5
- Female *Asaia krungthepensis* sample1
- Female *Asaia krungthepensis* sample2
- Female *Asaia krungthepensis* sample3
- Female *Asaia krungthepensis* sample4
- Female *Asaia krungthepensis* sample5
- Female *Asaia bogorensis* sample1
- Female *Asaia bogorensis* sample2
- Female *Asaia bogorensis* sample3
- Female *Asaia bogorensis* sample4
- Female *Asaia bogorensis* sample5
- Water (negative control)
- Positive control *Asaia krungthepensis*

- Positive control *Asaia bogorensis*
- Promega 1kb DNA ladder
- Invitrogen 100bp DNA ladder

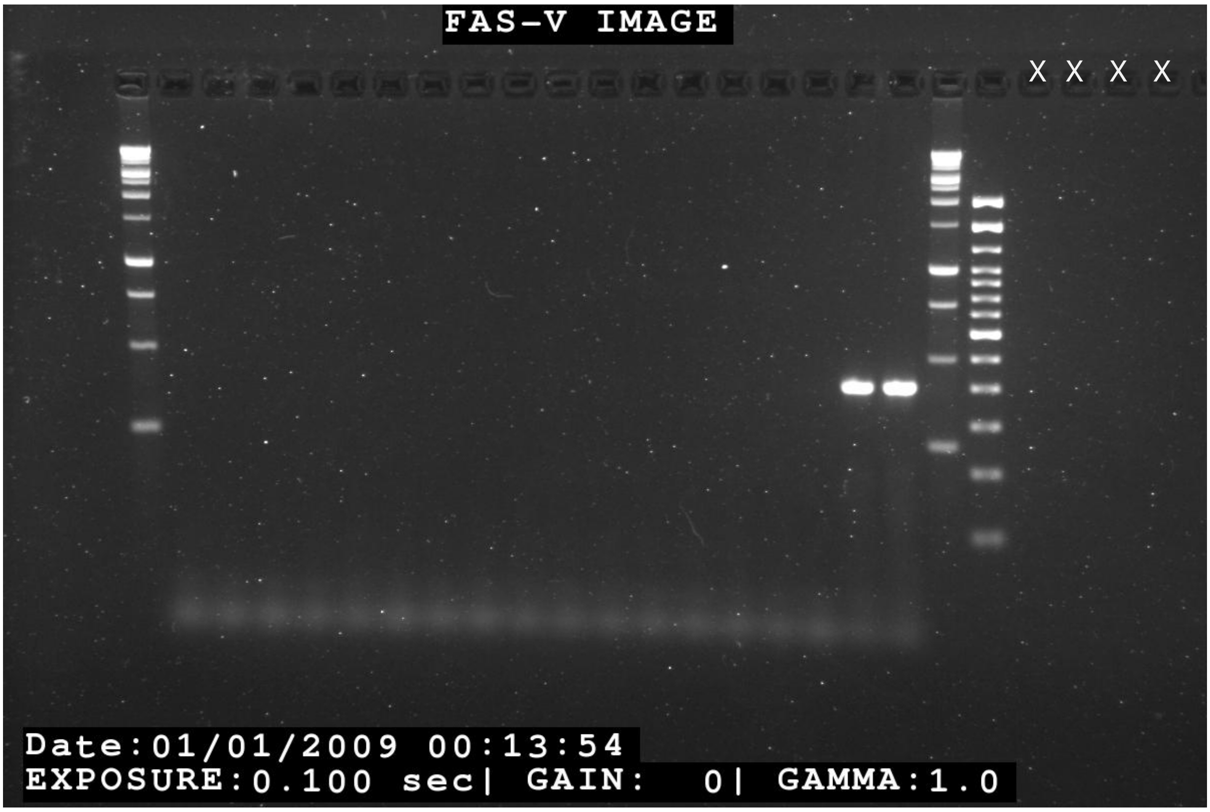

Fig 7.B) Right panel: PCR for the detection of *Asaia* spp. in adult females. *Culex* specific primers were used as positive control of DNA extraction and PCR procedure.

From left to right:

PCR with *Cx. Pipiens* specific primers (CQ11F2 (5' - GATCCTAGCAAGCGAGAAC) and pipCQ11 r (5' - CATGTTGAGCTTCGGTGAA)):

- Promega 1kb DNA ladder
- Female Control sample1
- Female Control sample2
- Female Control sample3
- Female Control sample4
- Female Control sample5
- Female *Asaia krungthepensis* sample1
- Female *Asaia krungthepensis* sample2
- Female *Asaia krungthepensis* sample3
- Female *Asaia krungthepensis* sample4
- Female *Asaia krungthepensis* sample5
- Female *Asaia bogorensis* sample1
- Female *Asaia bogorensis* sample2
- Female *Asaia bogorensis* sample3

- Female *Asaia bogorensis* sample4
- Female *Asaia bogorensis* sample5
- Water (negative control)
- Positive control *Asaia krungthepensis*
- Positive control *Asaia bogorensis*
- Promega 1kb DNA ladder
- Invitrogen 100bp DNA ladder

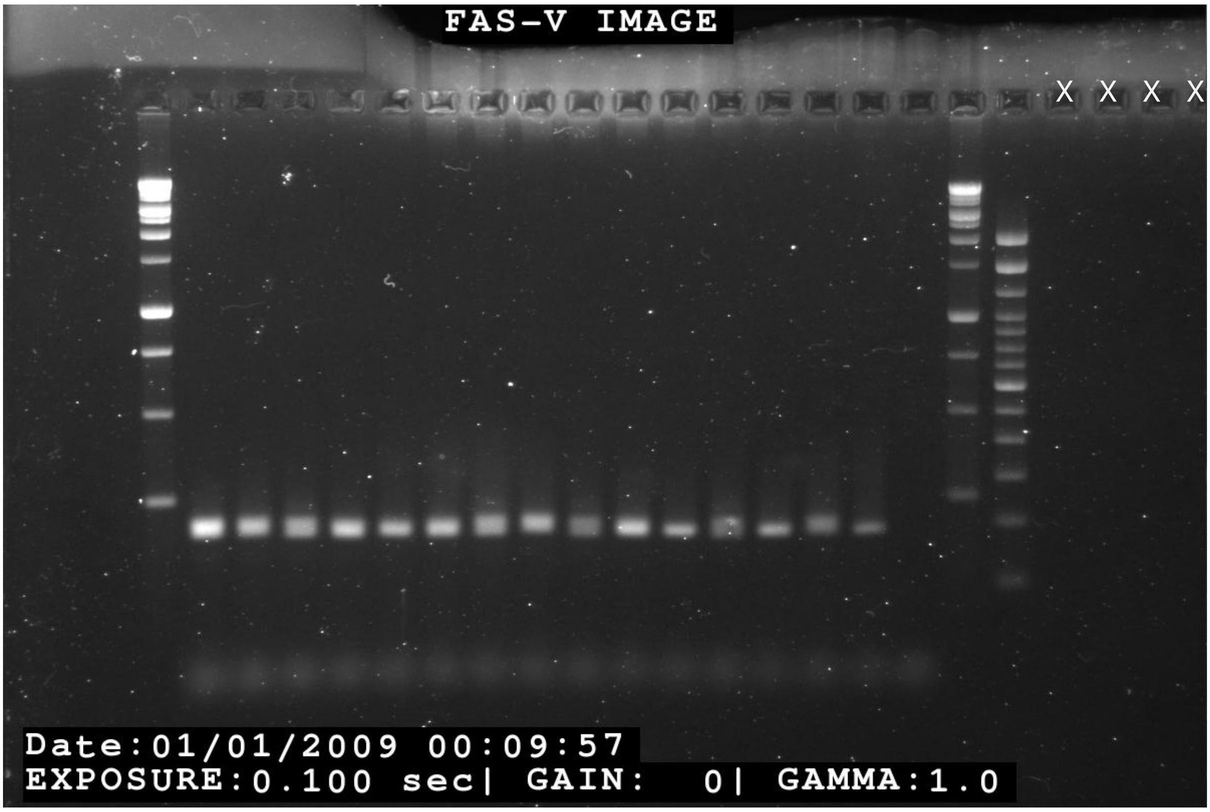

Supplement: S1 Data — (PDF) [file pone.0330703.s001.pdf]
